# Adaptive Feedback Control in Human Reaching Adaptation to Force Fields

**DOI:** 10.3389/fnhum.2021.742608

**Published:** 2021-12-27

**Authors:** James Mathew, Frédéric Crevecoeur

**Affiliations:** ^1^Institute of Information and Communication Technologies, Electronics and Applied Mathematics (ICTEAM), Catholic University of Louvain, Louvain-la-Neuve, Belgium; ^2^Institute of Neuroscience (IoNS), Catholic University of Louvain, Louvain-la-Neuve, Belgium

**Keywords:** feedback control, motor adaptation, reaching control, sensorimotor integration, computational models

## Abstract

Sensorimotor adaptation is a central function of the nervous system, as it allows humans and other animals to flexibly anticipate their interaction with the environment. In the context of human reaching adaptation to force fields, studies have traditionally separated feedforward (FF) and feedback (FB) processes involved in the improvement of behavior. Here, we review computational models of FF adaptation to force fields and discuss them in light of recent evidence highlighting a clear involvement of feedback control. Instead of a model in which FF and FB mechanisms adapt in parallel, we discuss how online adaptation in the feedback control system can explain both trial-by-trial adaptation and improvements in online motor corrections. Importantly, this computational model combines sensorimotor control and short-term adaptation in a single framework, offering novel perspectives for our understanding of human reaching adaptation and control.

## Introduction

Sensorimotor adaptation can be characterized by an update of motor commands following changes in body or environment dynamics. This critical function of the nervous system allows humans and other animals to improve the efficiency of their movements with practice. Traditionally, studies on upper limb reaching movements in laboratory settings have described trial-by-trial improvement performance in terms of two interacting processes: *feedforward* and *feedback* control. Feedforward control can be defined as the formation of motor commands independent of sensory feedback, and it is typically associated with predictive aspects and planning. Feedback control refers to real-time adjustments of motor commands based on sensory inflow. These two controllers can be modified between movements (i.e., offline) or within a movement (online). Characterizing how adaptation impacts feedforward and feedback control has recently been a lively research topic.

In the context of force field learning, it is assumed that the difference between actual and expected sensory information, also called sensory prediction error, is used internally to re-calibrate an internal model of limb and environment dynamics (Shadmehr et al., 2010; Wolpert et al., 2011). Here, we adopt a generic definition of an internal model, as a neural mechanism that can simulate the consequences of an action, and drive estimation and control based on this knowledge (McNamee and Wolpert, 2019). Accordingly, adaptation has often been understood as an iterative update of the feedforward controller following sensory prediction errors from the previous movement. Recently, mounting evidence has highlighted that adaptation was not confined to the feedforward process, as it also occurs in the feedback control system. Yet a theory linking feedforward and feedback adaptation has been lacking.

Here, we present computational models of reaching adaptation and review current evidence that adaptation also impacts feedback control. We highlight that the feedback control system can adapt without necessarily implying changes in behavioral proxies of feedforward control such as initial movement directions. Moreover, evidence suggests that adaptation in feedback pathways can occur within a time interval shorter than the time of a reaching movement, which is difficult to reconcile with a sequential adaptation of feedforward and feedback controllers across trials. These observations suggest that models of sensorimotor adaptation require revision to include adaptation in feedback pathways explicitly. We describe a candidate model to accommodate these behavioral findings.

## Computational Models of Human Reaching Adaptation

Models of human reaching adaptation have typically dissociated trial-by-trial changes in movement performances from continuous variables that the nervous system handles within a movement, thereby separating control and adaptation mechanisms. A standard definition of a trial is a single point-to-point movement, but it is clear that this artificial construct has impacted models of adaptation and that translating the concepts developed below to continuous tasks, such as cyclic movements or tracking, is an important question for prospective work. Although it is accepted that adaptation is a continuous process (Krakauer et al., 2019), the main computational models characterize discrete-time adaptation with a time step is equal to a trial. The categories presented below also correspond to model properties, which are not exclusive, thus some previously published models fall into several categories.

A first category corresponds to *time-series* models, which aim at capturing the evolution of learning curves across trials. A prominent example is the two-states model proposed by Smith and colleagues (Smith et al., 2006), who demonstrated that there exist fast and slow processes that learn and forget at different rates. Kording et al. (2007), added that multiple timescales could underlie the dynamics of memory. The addition of multiple timescales was also associated with a parallel architecture in the context of visuomotor adaptation (Lee and Schweighofer, 2009). Although these models differ by their structure, they make the same assumption that any error [or filtered error (Wei and Körding, 2010)] perceived on a given trial influences the next trial. Indeed, in Smith et al. (2006), the time unit was the trial. In Kording et al. (2007), it was hypothesized explicitly that the fastest timescale in the adaptation model was slower than the movement time, thereby only allowing trial-by-trial adjustments. These models also describe the evolution of an abstract state variable (or *motor gain*), without considering continuous variables related to movement execution, hence it is it difficult to link adaptation and control in this framework.

A second category of adaptation models can be referred to as *partial compensation*. Contrary to time-series models, these models express a control problem in continuous time with partial knowledge of environment dynamics. For instance, Shadmehr and Mussa-Ivaldi (1994) used a model based on trajectory tracking with an adaptive internal model. Mistry and colleagues (Mistry et al., 2013) made similar assumptions in the context of Linear-Quadratic-Gaussian (LQG) control (Todorov and Jordan, 2002), with an estimated plant dynamics that differed from the true plant dynamics including the force field. Recently, Ikegami and colleagues (Ikegami et al., 2021) used the same approach to demonstrate that both target failure and altered hand path may interact to drive adaptation hierarchically. In these models, the level of adaptation depended on how much the force field was compensated during movement by the approximate internal model, which simply takes the form of a function used in the controller. While they explicitly formulated a control problem in continuous time, these models did not include any learning rule that transforms sensory mediated errors into a novel model estimate for the next movement.

A relationship between discrete-time adaptation and continuous control can be found in the following classes of models. The first includes *motor primitives* as building blocks linking continuous control during a movement and updates between movements. Motor primitives are defined as basis functions available in the brain tuned to position and velocity (Thoroughman and Shadmehr, 2000; Hwang et al., 2003), which are combined to minimize the error between actual and ideal or expected forces. In this framework, the internal model takes the form of a weighting matrix used to combine the primitives. The power of this theory has been to capture human generalization patterns. The main question toward linking adaptation and control with motor primitives is whether this model can reproduce behaviorally the same properties as state-feedback controllers, which characterize human motor responses to perturbations (Crevecoeur and Kurtzer, 2018).

In favor of this idea, Sing and colleagues (Sing et al., 2013) argued that limb motion determined adaptation independent of the disturbance profile, suggesting that the variables underlying adaptation are limb position and velocity. However, this hypothesis is at odds with the fact that similar patterns of motion evoke different feedback responses dependent on the limb configuration and context, suggesting that an internal model of limb dynamics and externally applied loads are used in the brain for online control (Kurtzer et al., 2008, 2009; Crevecoeur and Scott, 2013; Maeda et al., 2017). Moreover, assuming that the weighting matrix of motor primitives can be used with time varying signals, it could be taken for a linear feedback controller. But in this case, this model does not include time-varying control gains known to characterize goal-directed reaching control in human (Liu and Todorov, 2007; Dimitriou et al., 2013; Poscente et al., 2021). Hence, the possibility that adaptation rests on the combination of motor primitives tuned to position and velocity may not capture all properties of human state-feedback control. The question arises as to whether it is still biologically plausible to assume the existence of a library of primitives including a broader set of variables as well as time-varying mixing matrices.

The other class of discrete time models: *adaptive impedance-control*, proposes muscle co-activation as a link between feedforward adaptation and online movement execution. According to this view, it is proposed that trial-by-trial adjustments were complemented by within-trial rejection of disturbances, inherent during early phases of adaptation, mediated by the limb intrinsic properties (Shadmehr and Mussa-Ivaldi, 1994; Franklin et al., 2003, 2008). In Shadmehr and Mussa-Ivaldi (1994), it was hypothesized that disturbances are countered by instantaneous opposition to deviation in position and velocity. Franklin and colleagues (Franklin et al., 2008) further demonstrated that trial-by-trial adjustments could be captured by changes in co-activation following unexpected muscles stretches (“V-shape” learning rule), while limb stability during movement was preserved in the model by the muscles viscoelastic properties. This model featured a simple learning rule, but the main shortcoming was that common estimates of limb stiffness are strongly impacted by feedback components. Indeed, measurements of stiffness are calculated up to ∼100 ms after an abrupt limb displacement (Burdet et al., 2000, 2001), thereby including proprioceptive, visuomotor, and early voluntary responses (Scott, 2016). Consequently, the relationship between online control and movement adaptation remains elusive.

To summarize, current computational models have in common the assumption that control during a movement is performed with a fixed internal model, and that adjustments are performed between two trials based on an error signal coming from the previous trial. In this view, it is easy to consider motor adaptation as an update in a feedforward pass across two trials. However, as we review in the next section, the expression of adaptation in feedback control makes feedforward and feedback adaptation mechanisms increasingly difficult to dissociate.

## Adaptation in Human Feedback Control System

Assuming separate forward and feedback passes with the adaptation of the feedforward pathway only can now be rejected in light of compelling evidence that adaptation of reaching movements also evokes changes in feedback control. A seminal study by Bhushan and Shadmehr proposed to include internal models in feedforward and feedback pathways (Bhushan and Shadmehr, 1999). Wagner and Smith (2008) demonstrated that resisting or assisting forces applied after adaptation to a lateral velocity-dependent force field evoked feedback responses with a lateral force component, indicative that the online correction took into account the acquired knowledge of the force-field. Subsequent studies showed that exposure to a force field evoked a modulation of visuomotor (Franklin et al., 2012), and long-latency pathways, that is as early as ∼60 ms following an abrupt load applied to the limb (Ahmadi-Pajouh et al., 2012; Cluff and Scott, 2013; Maeda et al., 2018). Long-latency responses have played a key role in understanding the neural basis of feedback control since they include a transcortical pathway through primary sensorimotor areas, premotor cortex, parietal areas, and cerebellum (Flament et al., 1984; Pruszynski et al., 2011; Omrani et al., 2016). Hence, it could be deduced from a modulation in long-latency responses that the underlying neural structures have access to the acquired knowledge of the force field.

It was further shown that changes in long-latency feedback gains paralleled the learning curve and correlated with the extent of adaptation (Cluff and Scott, 2013). More recently, a modulation in long-latency feedback gains has been linked to the fast time-scale of movement adaptation in a dual-rate model (Coltman and Gribble, 2020). A comparable change in long-latency response gain has been associated with transient and unpredictable disturbances, evoking co-contraction and modulation of overall control gains (Crevecoeur et al., 2019). It remains unclear when changes in long-latency responses start expressing knowledge of the new force field rather than reflecting a robust control strategy. But clearly, over the course of a few trials, the imprint of movement errors in the brain produces adjustments in the neural bases of both feedforward and feedback controllers.

**FIGURE 1:**
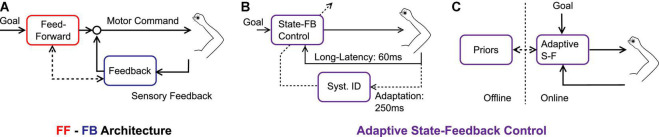
**(A)** Feedforward-feedback control architecture, each pass corresponds to different neural structures that share knowledge of the environmental dynamics. Feedforward and Feedback are associated with red and blue colors, respectively. **(B)** Adaptive state-feedback control model in which an identification of the system parameters (Syst. ID) updates a state-feedback controller online (State-FB Control). The timescales are represented: it is assumed that state-feedback control is supported by long-latency feedback loops (timescale: ∼60 ms), and the online updates are associated with a slower timescale (∼250 ms). **(C)** Conceptual representation of the adaptive state-feedback controller which can replace feedforward and feedback mechanisms, while different time scales are associated with online and offline mechanisms.

These previous studies still implicitly assumed that feedforward adaptation occurred and the feedback control system inherited or shared the novel reach representation to produce adapted feedback responses. However, there is also evidence that adaptation occurs in the feedback control system without adapting the feedforward mechanism. Indeed, Maeda and colleagues (Maeda et al., 2020) trained volunteers to counter perturbation while blocking shoulder motion physically. They observed that participants reduced their shoulder response, which in turn affected reaching movements performed when the shoulder was suddenly unlocked. Thus, internal representations of dynamics (in this case, the limb dynamics) could be acquired by exposing the feedback control system only. It is therefore necessary to at least consider adaptation in both feedforward and feedback pathways with reciprocal interactions (Figure 1A). An additional property must still be added to the picture: the possibility that adaptation occurs online, within a movement, as suggested in our recent series of reports.

Indeed, we documented evidence for adaptation in feedback responses to unpredictable application of a force field during reaching. By looking at the whole movement execution, we showed that participants learned to correct for the unexpected disturbance without anticipation, and that the tuning of the force profiles applied by participants to the handle, displayed the same properties as adapted movements measured in a standard adaptation paradigm (Crevecoeur et al., 2020b). The possibility that movement representation was different at the beginning (no anticipation) and end (adapted feedback) of a single reaching movement means that the controller changed online. The improvement in feedback corrections was observed for force fields of different directions and different kinds, and was expressed while relearning to move in a previously experienced force field (Crevecoeur et al., 2020a; Mathew et al., 2021). These responses also evoked rapid and stable re-planning when they were performed in a rapid sequence of movements (Mathew et al., 2020).

Measuring the timescale of this process was crucial. When we trained participants to perform a rapid movement including a stop-over at a via point, we observed that the second movement, from the via-point to the goal, was quickly updated according to a force field perturbation experienced before the via-point. This update was visible in hand kinematics as early as ∼0.5 s following reach onset (Crevecoeur et al., 2020b; Mathew et al., 2020). In a different experiment, we observed that adaptive changes in muscles recordings that correlated with force modulation occurred after ∼250 ms following reach onset (Crevecoeur et al., 2020a). Thus, the timescale of adaptation may lie between 250 ms (from EMG) and 500 ms (from hand kinematics).

Including such a fast timescale of adaptation in a computational model of reaching control is a two-sided story: on the one hand, there is no difference with previous models since it also considers that sensory prediction errors update internal representations. On the other hand, the fact that adaptation happens faster than a trial blurs the distinction between feedforward and feedback mechanisms.

## Adaptive State-Feedback Control Model

The candidate model to explain the forgoing observations was based on adaptive state-feedback control (Bitmead et al., 1990). It must be noted that the computational advantage of an adaptive neural controller was first discussed by Fortney and Tweed (2011). The basic premise is that the state-feedback controller is parameterized based on knowledge of the limb and environment dynamics, coupled with an identification procedure that can change the parameters of the controller online (Figure 1B). The model can be viewed as two nested loops: the state-feedback controller describes how the nervous system responds to changes in state variables for a fixed parameterization, and the adaptive loop consists in online tuning of the model parameters. When mapped onto human neural mechanisms, we submit that the state-feedback control loop is mediated by long-latency circuits (∼60 ms) (Crevecoeur and Kurtzer, 2018), while adaptation is associated with a longer timescale (>250 ms, Figure 1B).

This model is very close to the standard view of human reaching adaptation while offering novel perspectives. In theory, the learning rate must not be too high, but there is no lower bound on the timescale at which the controller can be re-parameterized. Thus it accommodates adaptation in real time and within a reaching movement. Second, the learning rule corresponds to a standard gradient descent: at each time step the parameter estimate makes a step in the direction that reduces the difference between expected and actual sensory input. It is of course a strong assumption to state that the nervous system performs a kind of gradient descent, however, this assumption is inherited from even the simplest learning models that make a step proportional and away from an error signal. It is the same learning rule as in previous models based on motor primitives, but it is applied to different variables. In the framework of motor primitives, the difference between sensed and expected forces (or trajectory) is used to change the mapping between primitives and force output, whereas in the framework of adaptive control, the difference between actual and expected sensory input is used to update a parametric representation of the system dynamics.

Importantly, the variables used to update the model are not abstract variables, such as learning states, instead they are the same state-variables as those assumed by the controller, i.e., neural encoding of joint angles, velocities, torques, muscles state, and potentially higher order derivatives. Thus, if we assume that these variables are used for control, we do not add complexity by assuming that they are also used for adaptation.

The adaptive feedback control model bridges together discrete-time models, and control models with partial compensation, simply by assuming that the time unit of adaptation is smaller than reaching time. This consideration suggests that the function of motor adaptation is not only to support changes in internal models over medium to long-term horizons but also to complement state-feedback control online. Hence, instead of considering separate feedforward and feedback processes (Figure 1A), we suggest that it is more accurate to consider online and offline mechanisms (Figure 1C). The online mechanism is an adaptive state-feedback controller. There is a daily life example of this mechanism: the adjustment of grip force that follows from lifting an unexpectedly heavy or light object. In this case, the object mass is a model parameter that is used to select control, and errors about this parameter produce not only feedback corrections but also changes in the parameter estimate. We propose that the same mechanism applies to online adaptation to velocity-dependent force fields. Other processes linked to consolidation and memory may work offline and follow longer timescales. Their expression takes the form of an internal prior, reflecting the expected dynamics during movement planning.

## Conclusion and Perspective

The adaptive feedback control model opens many questions and challenges. From a computational perspective, it is clear that non-linear dynamics and delays limit the range of feasible online adaptation rates. This theoretical limit is currently unknown and it may impact the generalizability of the model. Moreover, by adapting parameters online the adaptive feedback controller becomes a non-linear control model. A theoretical bound on the adaptation rate would also limit the range of non-linear effects that this model can handle. We believe that it offers the opportunity to understand which classes of movements can be handled with adaptive linear approximations and which movement properties require a forward pass to cope with non-linear effects.

Another clear challenge is to link adaptive feedback control with other learning mechanisms. We focused on adaptation to force fields, but evidence for online adaptation has been also reported with random visuomotor perturbations (Braun et al., 2009). Besides, there are different ways the nervous system expresses improvements in behavior including use-dependent learning, reinforcement learning, and explicit strategies (Krakauer et al., 2019). The relationship between adaptive state-feedback control and these different learning schemes remains to be established.

Finally, we believe that rapid feedback adaptation could become a behavioral proxy of fast neural learning mechanisms recently hypothesized (Sohn et al., 2021). On the one hand, changes in connectivity in a network model of sensorimotor adaptation may capture plasticity mechanisms and long-term adaptation, on the other hand rapid or online adaptation must rely on changes in neural trajectories for a fixed network configuration (Sohn et al., 2021). It is expected that the imprint of online adaptation is visible as changes in dimension or shape of neural trajectories associated with reaching control.

## Author Contributions

Both authors listed have made a substantial, direct, and intellectual contribution to the work, and approved it for publication.

## Conflict of Interest

The authors declare that the research was conducted in the absence of any commercial or financial relationships that could be construed as a potential conflict of interest.

## Publisher’s Note

All claims expressed in this article are solely those of the authors and do not necessarily represent those of their affiliated organizations, or those of the publisher, the editors and the reviewers. Any product that may be evaluated in this article, or claim that may be made by its manufacturer, is not guaranteed or endorsed by the publisher.
